# Halo Sport Transcranial Direct Current Stimulation Improved Muscular Endurance Performance and Neuromuscular Efficiency During an Isometric Submaximal Fatiguing Elbow Flexion Task

**DOI:** 10.3389/fnhum.2022.758891

**Published:** 2022-02-17

**Authors:** Lejun Wang, Ce Wang, Hua Yang, Qineng Shao, Wenxin Niu, Ye Yang, Fanhui Zheng

**Affiliations:** ^1^Sport and Health Research Center, Physical Education Department, Tongji University, Shanghai, China; ^2^Shanghai Yangzhi Rehabilitation Hospital, School of Medicine, Tongji University, Shanghai, China; ^3^College of Physical Education and Health Science, Yibin University, Sichuan, China; ^4^Shanghai Research Institute of Sport Science, Shanghai, China

**Keywords:** tDCS, halo sport, endurance performance, EEG, EMG

## Abstract

The present study examined the effects of transcranial direct current stimulation (tDCS) using Halo Sport on the time to exhaustion (TTE) in relation with muscle activities and corticomuscular coupling of agonist and antagonist muscles during a sustained isometric fatiguing contraction performed with the elbow flexors. Twenty healthy male college students were randomly assigned to tDCS group and control group. The two group participants performed two experimental sessions which consisted of pre-fatigue isometric maximal voluntary contraction (MVC), sustained submaximal voluntary contractions (30% maximal torque) performed to exhaustion, and post-fatigue MVC with the right elbow flexor muscles. Sham stimulation (90 s) and tDCS (20 min) were applied for control and tDCS group participants 20 min prior to the second session test, respectively. MVC strength in pre- and post-fatigue test, TTE, electroencephalogram (EEG), and electromyography (EMG) of biceps brachii (BB) and triceps brachii (TB) were recorded during the tests. It was found that tDCS using the Halo Sport device significantly increased TTE and thus improved muscular endurance performance. The improvement may be partly related to the improvement of neuromuscular efficiency as reflected by decrease of antagonistic muscle coactivation activities, which may be related to cortical originated central processing mechanism of neuromuscular activities.

## Introduction

Muscular endurance is the ability to continue contracting a muscle, or group of muscles against resistance over a long period of time, which describes the muscles’ ability to fight against fatigue locally during a longer exercise (Bigland-Ritchie and Woods, 1984). It is a health-related component of physical fitness as well as basic athletic ability in sports performance (Sleivert and Rowlands, 1996; Neumayr et al., 2003). Thus, exploring ergogenic aids to improve muscular endurance is of foremost concern (Schubert and Astorino, 2013). Particularly, in recent decades, researches have focused on the study of the brain as the central governor to regulate and improve the muscular endurance performance (Gandevia, 2001; Noakes, 2011, 2012).

Transcranial direct current stimulation (tDCS) is an emerging technique that has received increasing attention due to its potential impact on brain activity in both athlete and non-athlete populations (Machado S. et al., 2019). tDCS is a noninvasive electrical brain stimulation, in which a weak electrical direct current (up to 2 mA for tens of minutes) is applied over the scalp to induce prolonged changes in brain excitability persisting for long after the current has ceased (Nitsche and Paulus, 2000, 2001). It has claimed that tDCS may improve training effects and boost exercise performance. For example, a systematic review revealed that studies investigating the efficiency of tDCS on improving muscular strength have demonstrated positive effects of tDCS in 66.7% of parameters tested (Machado S. et al., 2019). However, with regards to muscular endurance, majority of current studies have demonstrated inconsistent influence and the effect of tDCS on muscular endurance performance remains elusive (Workman et al., 2020).

As there are multiple brain regions that may be involved in exercise regulation, the rationale for using tDCS for performance enhancement may vary accordingly, which may provide an explanation for the inconsistent results of different studies (Machado D. G. D. S. et al., 2019). It seemed that different stimulation conditions and parameters may contribute to different results (Bastani and Jaberzadeh, 2012). Moreover, warm up or training exercises conducted simultaneously with tDCS have been adopted in a few previous studies, which may be a factor to influence the results (Edwards et al., 2017; Park et al., 2019). Halo Sport device is a commercial system that consists of a headset similar to conventional headphones, which uses tDCS to deliver weak direct currents over the scalp through surface electrodes, termed primers, with the intention of inducing changes in both sides of the motor cortex (Huang et al., 2019). Halo Sport has been used in training and competition (Hornyak, 2017), but researches have rarely explored the effect of Halo Sport device on muscular endurance.

The objective of this work was to study the effects of tDCS using Halo Sport on the time to exhaustion (TTE) in relation with muscle activities and corticomuscular coupling of agonist and antagonist muscles during a sustained isometric fatiguing contraction performed with the elbow flexors. With reference to relevant previous researches, we have adopted the setup of dynamic elbow flexion exercise combined with 20-min tDCS. We hypothesized that tDCS using Halo Sport may exert significant effect on isometric muscular endurance performance of elbow flexors associated with changes of muscle activities and intramuscular coupling of agonist and antagonist muscles.

## Materials and Methods

### Participants

In this study, women were excluded in order to control for hormonal fluctuations and their effect on cortical excitation (Rudroff et al., 2020). As a result, a total of 20 healthy college students (men) with dominant right arm were randomly assigned to tDCS group and control group. The age of the participants ranged from 18 to 25 years. The physical characteristics of the two group participants are listed in Table 1. Participants were asked to refrain from exercising and consuming alcohol or caffeine 24 h before the experimental sessions. The participants did not report neurological disorders or upper limb musculoskeletal diseases and had not engaged in regular exercise training of their upper limbs for at least 12 months. All the participants gave written informed consent in accordance with the Declaration of Helsinki and participated voluntarily. The experiment was approved by the Ethics Committee of Tongji University.

**TABLE 1:** Physical characteristics of the four group participants.

	*N*	Age (year)	Height (cm)	Weight (kg)	BMI
tDCS group	10	20.6 ± 4.1	177.9 ± 4.5	66.4 ± 7.6	21.0 ± 2.5
Control group	10	21.4 ± 2.4	178.4 ± 9.4	71.4 ± 12.9	22.3 ± 3.0

### Data Recording

#### Experimental Procedure

Each participant visited the laboratory on two different occasions. During the first visit, participants were familiarized with all experimental equipment and procedures. In addition, participants completed an isometric maximum voluntary contraction (MVC) test with the right elbow flexor muscles for the determination of maximal elbow flexion torque. Three attempts of MVC were performed, separated by 5 min, and the best of the three attempts was chosen as MVC (Bazzucchi et al., 2008). Then participants were asked to sit comfortably in a chair in an upright position with the upper arm vertically placed, and the elbow angle was maintained at 90°. The right forearm was positioned parallel to the ground and supinated. The participants were instructed to maintain their posture by flexing the elbow at a target value of 30% MVC torque as close as possible to 90° until they experienced exhaustion and were no longer able to continue the contraction. The criteria for stopping the muscle fatigue testing was that the angle of the elbow joint should be larger than 100° and last for more than 5 s. The participants were verbally vigorously encouraged to continue the sustained contraction for as long as possible. Surface electromyographic (EMG) signals from the right biceps brachii (BB) and triceps brachii (TB) muscles of the right limb and EEG were recorded simultaneously. The participants were instructed to perform another MVC test as soon as the fatiguing elbow flexion contraction task was complete.

**FIGURE 1:**
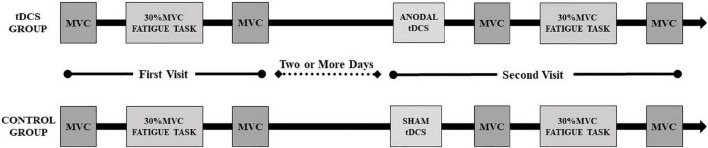
Overall view of the experimental protocol.

The experiments days were separated by two or more days to exclude fatigue of the experiment. All the sessions were performed at the same time of the day to minimize daily variability. In the second visit, the participants of the tDCS group received 20-min anodal stimulation while the control group received 20-min sham stimulation by Halo Sport (Halo Neuroscience Inc., San Francisco, CA, United States). During the 20-min stimulation, participants performed dynamic elbow flexion practice for eight sets with the right hand holding a 3 kg dumbbell (15 times/set, 3 s for one time and 1 s for two consecutive practice intervals). A 1-min rest interval was given between each set to refrain from muscle fatigue. Then the steps in the first visit were repeated. The overall view of the experimental protocol is presented in Figure 1.

#### Maximum Voluntary Contraction Measurement

The MVC of the right elbow flexion was tested at 90° of elbow flexion using a torque sensor (TRS-500; Transducer Techniques Inc., Temecula, CA, United States). The sensor was located in line with the center of the rotation of the active elbow joint. The participants were verbally encouraged to perform three MVCs with 5 s duration, each separated by 5-min. The highest of the three MVCs was used as the MVC value (Bazzucchi et al., 2008).

#### Electroencephalogram Measurement

Electroencephalogram was recorded continuously during the sustained contraction from Fz, C3, Cz, C4, and Pz using a scalp of 64-channel NeuroScan SynAmps system (NeuroScan Inc., El Paso, TX, United States). We have done head measurements and used the 10–20 EEG convention to determine the location of Cz as well as other EEG electrodes. The impedance for all electrodes was monitored and settled below 5 kΩ EEG signals were amplified (×75,000, NeuroScan SynAmps RT amplifier), band-pass filtered (0.01–100 Hz), digitized (2,000 samples/s), and acquired. The participants were required to concentrate on the task performance and minimize distractions as much as possible. They were asked to maintain a stable body position and avoid teeth biting and head movements during the sustained isometric elbow flexion contraction (Wang et al., 2017; Wang et al., 2020a).

#### Electromyography Measurement

Surface EMG signals were recorded by NeuroScan system (NeuroScan Inc., El Paso, TX, United States). Bipolar electrode pairs were placed longitudinally over the right BB and TB muscle bellies at an inter-electrode distance of 2 cm according to SENIAM recommendations^1^. A common reference electrode was placed on the left mastoid. Skin was shaved and cleaned with alcohol to minimize impedance before applying the electrodes. The electrodes filled with conducting gel were secured with surgical tape. The EMG signals were amplified (×1,000), band pass filtered (3∼1,000 Hz), and digitized (2,000 samples/s). All logged data were stored on a computer for further analysis.

#### Transcranial Direct Current Stimulation Procedure

The Halo Sport device has three studded foam electrodes (24 cm^2^/electrode) that can apply no more than 2.2 mA direct currents to the scalp to induce changes in both sides of the motor cortex. The electrodes were moistened with normal saline (0.9% NaCl) before the Halo Sport device was placed over the vertex of the head of each participant correctly. The electrical current was ramped up to 2.0 mA over the course of 30 s. In the tDCS group, the current was held at 2 mA for 20 min. In the control group, current was ramped down to 0 mA after 90 s. Participants were blind to the stimulation condition. After completing the experiment, participants stated that they were unable to tell whether tDCS or sham stimulation was applied.

### Data Processing and Analysis

#### Data Pre-processing

The EEG signals pre-processing procedure included re-referencing calculation (to the average value of the bilateral mastoids), ocular artifacts reducing, band pass filter at 3–60 Hz and artifact rejection (based on the criteria of signals exceeding ±100 μV at any time point). EMG signals were band pass filtered (5–500 Hz) using an FIR zero-phase-shift filter. The procedure was completed using Scan 4.3 software (NeuroScan Inc., El Paso, TX, United States). Finally, EMG and EEG signals of each participant were segmented to two equal-length parts: the 1st half and 2nd half of the contraction for the later analysis.

#### Electromyography Amplitude, Coactivation Ratio, and Median Frequency Calculation

The filtered EMG signals of the 1st and 2nd half contraction recorded from BB and TB were full wave rectified. Following full wave rectification, the EMG signals were root mean squared with a 100 ms moving rectangular window to create a linear envelope. EMG root mean square (RMS) of each muscle and antagonist muscle coactivation levels were calculated based on the EMG envelope, while median frequency (MF) were calculated based on Fourier transform of raw EMG signals. Calculation methods were referenced to the research of Wang et al. (2015) and were performed for non-overlapping 2.048-s duration epochs.

#### Electromyographic–Electroencephalogram Phase Synchronization Analysis

For each participant, EMG and EEG signals recorded in isometric sustained contraction were divided into the 1st half and 2nd half of the contraction as described above. The selected EMG and EEG data were filtered for the frequency range 8–12 Hz (alpha band), 15–35 Hz (beta band) and 35–60 Hz (gamma band) by means of a 4th order zero-phase-shift Butterworth filter. Phase synchronization analysis was then conducted and phase synchronization index (PSI) of between EMG and EEG was calculated as (Quiroga et al., 2002)


(1) $$P\,S\,I=\sqrt{{\left\langle c\,o\,s\,\theta_{x\,y}^{H}\,\left(t\right)\right\rangle }_{t}^{2}+{\left\langle s\,i\,n\,\theta_{x\,y}^{H}\,\left(t\right)\right\rangle }_{t}^{2}}$$

where ⟨_._⟩*_t_* means the average of all the values and


(2) $$\theta_{x\,y}^{H}\,\left(t\right)=n\,\theta_{x}^{H}\,\left(t\right)-m\,\theta_{y}^{H}\,\left(t\right)$$

in which $\theta_{x}^{H}\,\left(t\right)$ and $\theta_{y}^{H}\,\left(t\right)$ are the phase angles calculated based on the Hilbert transform of EMG and EEG. The value of *m* and *n* were all assigned to 1 (Quiroga et al., 2002).

For each muscle, the PSI calculated between the EMG and EEG of Fz, C3, Cz, C4, Pz were averaged and the average EMG–EEG PSI of each BB and TB muscle were acquired. Data processing was performed using the MATLAB 2009Ra software (MathWorks Inc., Natick, MA, United States).

### Statistical Analysis

Statistical analysis was performed using the SPSS 13.0 for Windows (SPSS Inc., Chicago, IL, United States). Normality was tested by means of the Kolmogorov–Smirnov test. A two-factor repeated-measures analysis of variance (between factors: tDCS group vs. control group; within factors: baseline vs. post-stimulation) was used to compare the contraction duration time. A three-factor repeated-measures analysis of variance (between factors: tDCS group vs. control group; within factors: baseline vs. post-stimulation; pre-fatigue vs. post-fatigue, or the 1st half contraction vs. the 2nd half contraction) was used to compare the maximal torque, BB, and TB EMG RMS (% MVC). Multiple comparisons with the Tukey’s *post hoc* tests were used to determine differences among pairs of means. Wilcoxon signed-rank test was used to test the difference of PSI between the 1st and 2nd half of contraction and between baseline and post-stimulation in alpha (8–12 Hz), beta (15–35 Hz) and gamma (35–60 Hz) frequency bands as PSI values have been examined to be non-normally distributed. The Pearson cross correlation analysis was used to observe the correlation between EMG median frequency and contraction duration time of the sustained fatiguing elbow contraction. All significance thresholds were fixed at α = 0.05.

## Results

### Contraction Duration Time and Maximal Torque

**FIGURE 2:**
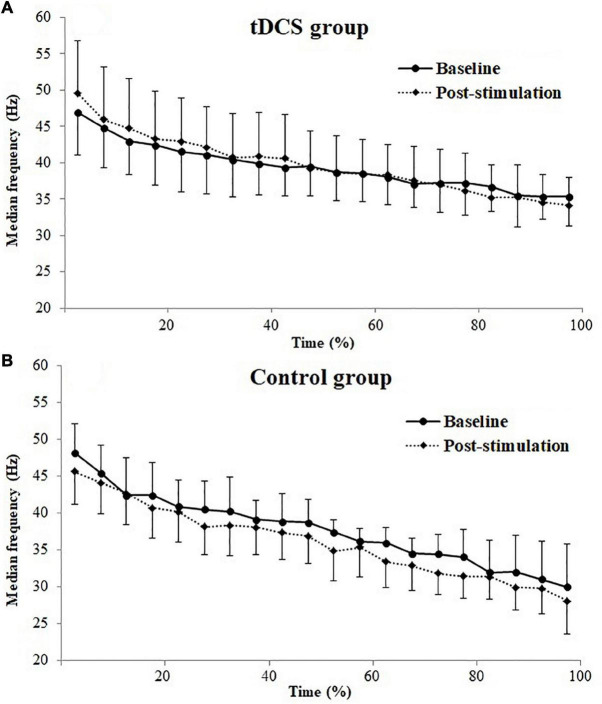
Electromyography (EMG) median frequencies (mean ± SD) of biceps brachii (BB) muscle for transcranial direct current stimulation (tDCS, **A**) and control **(B)** group subjects plotted as a percentage of contraction time during the sustained fatiguing elbow flexion contraction in baseline and post-stimulation test.

Figure 2 represents the changes of EMG median frequency of BB muscle during the sustained fatiguing elbow flexion contraction in baseline and post-stimulation test. A progressive reduction in the EMG median frequency was observed in both the tDCS and control group participants in baseline and post-stimulation test, during the fatiguing contraction. Significant positive correlations between EMG median frequency and contraction duration time were observed in both tDCS and control group participants in baseline and post-stimulation test (tDCS group × baseline: *r* = −0.643, *P* = 0.000; tDCS group × post-stimulation: *r* = −0.613, *P* = 0.000; control group × baseline: *r* = −0.855, *P* = 0.000; control group × post-stimulation test: *r* = 0.888, *P* = 0.000).

**FIGURE 3:**
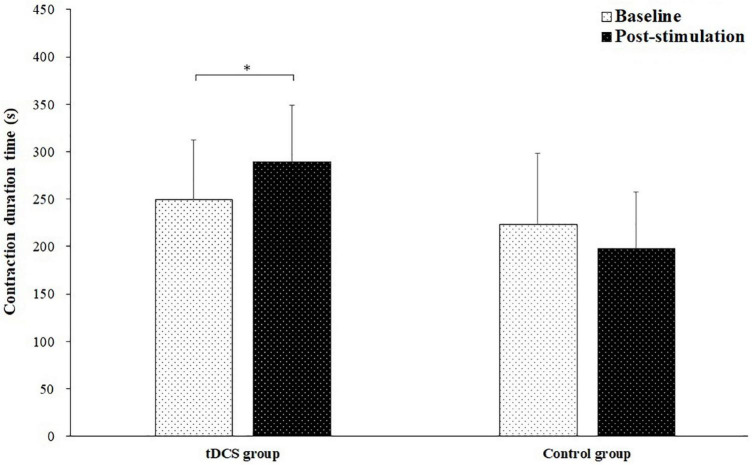
Contraction duration time (mean ± SD) of the sustained fatiguing elbow flexion contraction for transcranial direct current stimulation (tDCS) and control group subjects in baseline and post-stimulation test. *demonstrated a significant difference of observed index between the baseline and post-stimulation test.

Figure 3 shows the contraction duration time of the sustained fatiguing elbow flexion contraction for tDCS and control group subjects in baseline and post-stimulation test. The contraction duration time of sustained fatiguing elbow flexion contraction during baseline and post-stimulation were 249.78 ± 62.33 and 289.33 ± 74.70 s for tDCS group, and 223.50 ± 59.79 and 197.90 ± 59.93 s for control group. There was a significant main effect of group (tDCS group vs. control group) on contraction duration time (*F* = 5.089, *P* = 0.037, partial eta-squared = 0.220). Moreover, a significant group by stimulation interaction effect was found (*F* = 10.786, *P* = 0.004, partial eta-squared = 0.375). It was found that the contraction duration time of tDCS group in post-stimulation test was significantly increased compared to baseline test (*P* = 0.030). There was no significant difference of contraction duration time between the baseline and post-stimulation test in control group (*P* = 0.072).

**FIGURE 4:**
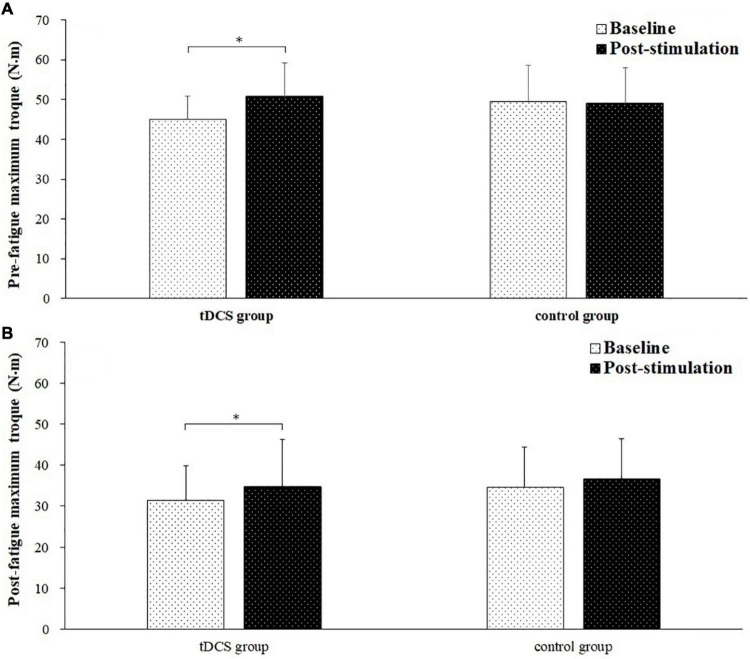
Pre- and post-fatigue maximal torque (mean ± SD) of elbow flexion tested during baseline and post-stimulation for transcranial direct current stimulation (tDCS) group and control group subjects. *demonstrated a significant difference of observed index between the baseline and post-stimulation test.

Figure 4 depicted the pre- and post-fatigue maximal torque of elbow flexion tested during baseline and post-stimulation for tDCS group and control group subjects. The pre-fatigue maximal torques during baseline and post-stimulation were 44.91 ± 5.89 and 50.71 ± 8.48 N m for tDCS group, and 49.32 ± 9.31 and 48.91 ± 9.05 N m for control group. The post-fatigue maximal torques were 31.56 ± 8.37 and 34.83 ± 9.55 N m for tDCS group, and 34.72 ± 11.48 and 36.67 ± 9.77 N⋅m for control group. There was a significant main effect of fatigue (*F* = 23.002, *P* = 0.000, partial eta-squared = 0.397) and stimulation (baseline vs. post-stimulation, *F* = 12.512, *P* = 0.001, partial eta-squared = 0.263) on maximal torque. Besides, a significant group by stimulation interaction effect on maximal torque has also been found (*F* = 6.264, *P* = 0.017, partial eta-squared = 0.152). The significant increase of maximal torque was only found in tDCS group after stimulation for both pre- (*P* = 0.006) and post-fatigue (*P* = 0.029) test. No significant difference of pre- (*P* = 0.768) and post-fatigue (*P* = 0.257) maximal torque was found between baseline and post-stimulation test for control group subjects.

### Agonist and Antagonist Muscle Activation Levels

**FIGURE 5:**
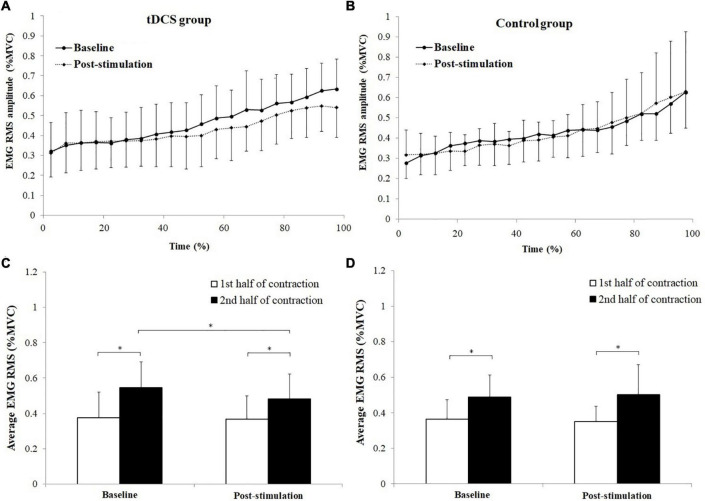
Changes of biceps brachii (BB) muscle activation levels during the sustained fatiguing elbow flexion contraction **(A,B)** and comparisons of average electromyography (EMG) root mean square (RMS) [% maximum voluntary contraction (MVC)] between the 1st and 2nd half of contraction for baseline and post-stimulation test in transcranial direct current stimulation (tDCS) **(C)** and control **(D)** group subjects. Data have been expressed as mean ± SD values. *demonstrated a significant difference of observed index between the baseline and post-stimulation test or between the 1st and 2nd half of contraction.

The changes of BB muscle activation levels during the sustained fatiguing elbow flexion contraction for tDCS and control group subjects are shown in Figures 5A,B. Progressive increases of EMG RMS (%MVC) were observed in both tDCS and control group subjects in baseline and post-stimulation test, during the fatiguing contractions. Besides, it can be seen that since the 40% time point, the average value of the BB EMG (%MVC) activation level was lower in post-stimulation test than in the baseline test, while the values of BB EMG (%MVC) activation level during the sustained contraction in baseline test were close to the values in post-stimulation test for control group subjects (Figures 5A,B). The average EMG RMS (%MVC) value of tDCS group subjects in the 1st and 2nd half of contraction were 0.378 ± 0.145 and 0.548 ± 0.144 for baseline test, and 0.370 ± 0.132 and 0.484 ± 0.139 for post-stimulation test. The values of control group subjects were 0.364 ± 0.111 and 0.489 ± 0.122 for baseline test, and 0.351 ± 0.086 and 0.502 ± 0.170 for post-stimulation test. A significant main effect of fatigue (*F* = 74.996, *P* = 0.000, partial eta-squared = 0.806), as well as significant stimulation × fatigue × group (*F* = 5.37, *P* = 0.036, partial eta-squared = 0.229) interaction effects on EMG RMS (%MVC) were found. The EMG RMS (%MVC) in the 2nd half of contractions were all significantly increased compared to the 1st half of contraction in baseline and post-stimulation test for both tDCS and control group subjects (tDCS group in baseline and post- stimulation test: *P* = 0.000 and *P* = 0.005, control group in baseline and post-stimulation test: *P* = 0.000 and *P* = 0.000). Besides, it has been found that the EMG RMS (%MVC) of the 2nd half contraction in the post-stimulation test was significantly lower than in the baseline test in only the tDCS group subjects Figures 5C,D. The statistics results of (1st and 2nd half of contraction for tDCS group: *P* = 0.788 and *P* = 0.048; 1st and 2nd half of contraction for control group: *P* = 0.606 and *P* = 0.777).

**FIGURE 6:**
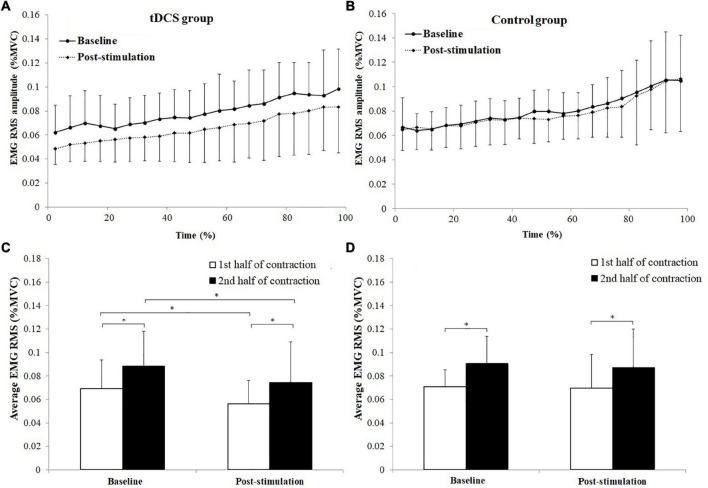
Changes of antagonist muscle coactivation level of triceps brachii (TB) during the sustained fatiguing elbow flexion contraction **(A,B)** and comparisons of average electromyography (EMG) root mean square (RMS) [% maximum voluntary contraction (MVC)] between the 1st and 2nd half of contraction for baseline and post-stimulation test in transcranial direct current stimulation (tDCS) **(C)** and control **(D)** group subjects. Data have been expressed as mean ± SD values. *demonstrated a significant difference of observed index between the baseline and post-stimulation test or between the 1st and 2nd half of contraction.

The changes of antagonist muscle coactivation level of TB during the sustained fatiguing elbow flexion contraction for tDCS and control group subjects are shown in Figures 6A,B. Progressive increases of TB EMG RMS (%MVC) were observed in both tDCS and control group subjects in baseline and post-stimulation test. Besides, it can be seen that the average value of the TB EMG (%MVC) activation level was lower in post-stimulation test than in baseline test only in tDCS group (Figures 6A,B).

The average TB EMG RMS (%MVC) value of tDCS group subjects in the 1st and 2nd half of contraction were 0.069 ± 0.024 and 0.088 ± 0.030 for baseline test, and 0.056 ± 0.020, 0.074 ± 0.035 for post-stimulation test. The average TB EMG RMS (%MVC) value of control group in the 1st and 2nd half of contraction were 0.071 ± 0.015 and 0.090 ± 0.023 for baseline test, and 0.070 ± 0.029 and 0.087 ± 0.033 for post-stimulation test.

A significant main effect of fatigue (*F* = 53.105, *P* = 0.000, partial eta-squared = 0.747), as well as significant stimulation × fatigue (*F* = 6.170, *P* = 0.023) and stimulation × fatigue × group (*F* = 5.94, *P* = 0.024, partial eta-squared = 0.323) interaction effects on TB EMG RMS (%MVC) were found. The TB EMG RMS (%MVC) in the 2nd half of contractions were all significantly increased compared to the 1st half of contraction in baseline and post-stimulation test for both tDCS and control group subjects (tDCS group in baseline and post-stimulation test: *P* = 0.000 and *P* = 0.01, control group in baseline and post-stimulation test: *P* = 0.000 and *P* = 0.004). Besides, it has been found that the EMG RMS (%MVC) in post-stimulation tests were significantly lower than in baseline tests for both the 1st and 2nd half of contraction in tDCS group rather than in control group subjects Figures 6C,D. The statistics results of (1st and 2nd half of contraction for tDCS group: *P* = 0.019 and *P* = 0.000; 1st and 2nd half of contraction for control group: *P* = 0.080 and *P* = 0.131).

### Neuromuscular Coupling

The Wilcoxon signed-rank test was used to test the difference of PSI between the 1st and 2nd half of contraction and between baseline and post-stimulation in alpha (8–12 Hz), beta (15–35 Hz), and gamma (35–60 Hz) frequency bands as PSI values have been examined to be non-normally distributed.

**FIGURE 7:**
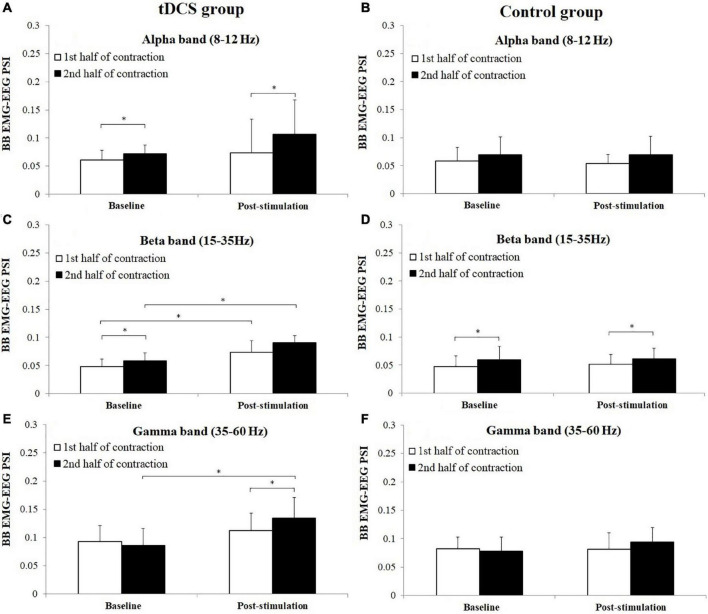
Comparisons of biceps brachii (BB) electromyography (EMG)–electroencephalogram (EEG) phase synchronization index (PSI) (mean ± SD) in alpha, beta, and gamma frequency bands between the 1st and 2nd half of contraction for baseline and post-stimulation test in tDCS **(A,C,E)** and control **(B,D,F)** group subjects. *demonstrated a significant difference of observed index between the baseline and post-stimulation test or between the 1st and 2nd half of contraction.

Figure 7 represents the average PSI in three frequency bands between BB EMG and EEG in tDCS and control group subjects. As PSI values were non-normally distributed for both group subjects of BB and TB muscles, which have been consistent with the distribution test result of PSI in previous research (Wang et al., 2015), the nonparametric Wilcoxon signed-rank test was used to test the difference of PSI between the 1st and 2nd half of contraction and between baseline and post-stimulation in alpha (8–12 Hz), beta (15–35 Hz), and gamma (35–60 Hz) frequency bands. For tDCS group, PSI in the 2nd half of contraction showed significant increase in alpha frequency band in baseline (*P* = 0.028, Cohen’s *d* = 0.68), and post-stimulation (*P* = 0.049, Cohen’s *d* = 0.55) test, beta frequency band in baseline test (*P* = 0.028, Cohen’s *d* = 0.77) test and gamma frequency in post-stimulation (*P* = 0.046, Cohen’s *d* = 0.62) test compared to the 1st half of contraction. For control group, significant increases of PSI were found in the 2nd half of contraction in beta frequency band in both baseline (*P* = 0.035, Cohen’s *d* = 0.57) and post-stimulation (*P* = 0.045, Cohen’s *d* = 0.24) test compared to the 1st half of contraction. Besides, it has been revealed that PSI in post-stimulation test were significantly higher in both the 1st (*P* = 0.047, Cohen’s *d* = 1.44) and 2nd (*P* = 0.007, Cohen’s *d* = 2.40) half of contraction in beta frequency band, and 2nd half of contraction in the gamma frequency band (*P* = 0.007, Cohen’s *d* = 1.41) compared to baseline test.

**FIGURE 8:**
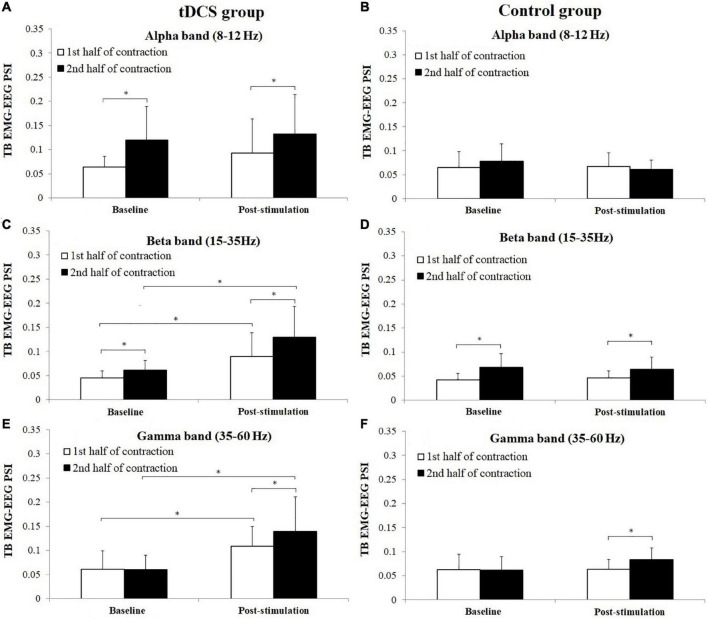
Comparisons of triceps brachii (TB) electromyography (EMG)–electroencephalogram (EEG) phase synchronization index (PSI) (mean ± SD) in alpha, beta, and gamma frequency bands between the 1st and 2nd half of contraction for baseline and post-stimulation test in tDCS **(A,C,E)** and control **(B,D,F)** group subjects. *demonstrated a significant difference of observed index between the baseline and post-stimulation test or between the 1st and 2nd half of contraction.

Figure 8 shows the average PSI in three frequency bands between TB EMG and EEG in tDCS and control group subjects. For tDCS group, PSI in the 2nd half of contraction showed significant increase in alpha frequency band in baseline (*P* = 0.028, Cohen’s *d* = 1.12) and post-stimulation (*P* = 0.047, Cohen’s *d* = 0.56) test, beta frequency band in baseline (*P* = 0.005, Cohen’s *d* = 0.93) and post-stimulation (*P* = 0.037, Cohen’s *d* = 0.70) test and gamma frequency in post-stimulation (*P* = 0.028, Cohen’s *d* = 0.55) test compared to the 1st half of contraction. For control group, significant increases of PSI were found in the 2nd half of contraction in beta frequency band in both baseline (*P* = 0.009, Cohen’s *d* = 1.14) and post-stimulation (*P* = 0.005, Cohen’s *d* = 0.90) test, and gamma frequency band in post-stimulation (*P* = 0.028, Cohen’s *d* = 1.58) test compared to the 1st half of contraction. Besides, it has been revealed that PSI of tDCS group in post-stimulation test were significantly higher in both the 1st and 2nd half of contraction in beta (1st half of contraction: *P* = 0.037, Cohen’s *d* = 1.32; 2nd half of contraction: *P* = 0.037, Cohen”s *d* = 1.47) and gamma frequency bands (1st half of contraction: *P* = 0.049, Cohen’s *d* = 1.29; 2nd half of contraction: *P* = 0.028, Cohen’s *d* = 1.56) compared to baseline test.

## Discussion

The present study examined the effects of tDCS using Halo Sport device on the TTE as well as muscle activities and corticomuscular coupling of agonist and antagonist muscles during a sustained isometric fatiguing contraction performed with elbow flexors. It has been found that tDCS with the Halo Sport device improved muscular endurance performance while exert significant influence on antagonist muscles activities. As far as we know, it is the first study to examine the effect of Halo Sport device on muscular endurance performance in relation with antagonist muscles adjustment.

In this study, maximal torque of elbow flexion was significantly increased as a result of tDCS in both pre- and post-fatigue conditions. The results were consistent with most of relevant previous researches in which significant muscle strength of MVC test were found after tDCS (Tanaka et al., 2009; Hendy and Kidgell, 2014; Frazer et al., 2017; Hazime et al., 2017; Vargas et al., 2018). The increase of maximal torque may indicate an improvement of neuromuscular system function to produce maximal muscle strength as a result of central modulation induced by tDCS, such as enhanced voluntary drives sent to the muscle, increases in corticospinal responsiveness and/or improved neuromuscular efficiency.

In previous researches, the effect of tDCS on muscular endurance has been widely investigated. However, majority of current studies have demonstrated inconsistent influence of tDCS on muscular endurance performance. A systematic review (Machado S. et al., 2019) of 13 relevant researches has found that seven studies revealed significant differences while no significant differences were found in six studies between tDCS and sham conditions under isometric contraction, muscle action against constant load, and isokinetic strength tests. Moreover, four articles examined the effects of tDCS on TTE (Angius et al., 2015; Vitor-Costa et al., 2015; Barwood et al., 2016; Lattari et al., 2018), two studies showed increases in endurance time after tDCS (Vitor-Costa et al., 2015; Lattari et al., 2018), while the other two studies did not reveal significant difference between tDCS and sham conditions (Angius et al., 2015; Barwood et al., 2016). It has been suggested that the different brain areas stimulated, stimulation conditions and parameters and other factors maybe contributed to the mixed results (Bastani and Jaberzadeh, 2012; Machado D. G. D. S. et al., 2019).

In previous researches, it has been suggested that the effect of tDCS on the enhancement of endurance performance may attribute to the increase of excitability of primary motor cortex (M1) and modulation of exercise-induced pain attenuation as a result of tDCS (Machado D. G. D. S. et al., 2019; Machado S. et al., 2019). However, it seemed that the improvement of exercise endurance performance may be partly explained by the improvement of neuromuscular efficiency as reflected by a significant decrease of antagonistic muscle coactivation in the current study, which has not been revealed by previous researches. The novelty of the results may be related to the specificity of tDCS adopted in this study. It has been suggested that tDCS may be effectively in motor practice and learning (Nitsche et al., 2007; Dumel et al., 2018), during which the adjustment of antagonistic muscle activities has been suggested as a training-specific response to increase exercise performance (Dutta et al., 2014; Wang et al., 2019). The results may indicate that tDCS may prompt obtainment of training gains by regulating activities of agonist and antagonist muscles.

Previous researches have demonstrated that oscillatory activity of the sensorimotor cortex shows coupling with muscle activity within the 15- to 35-Hz frequency band during weak to moderate sustained isometric contraction, whereas strong voluntary contractions are associated with coherence at gamma frequency band (Baker et al., 1997; Brown et al., 1998; Wang et al., 2020a). In this study, significant increases of EEG–EMG PSI in beta and gamma frequency band were found in both BB and TB muscles due to tDCS. The increases of EEG–EMG PSI may demonstrate enhanced coupling between cortical output centers associated with weak to strong voluntary contractions and peripheral muscles that received the cortical signals to carry out the intended motor action as a result of tDCS (Xi et al., 2021a,b). Therefore, the results revealed in this study demonstrate that cortical related central processing mechanism of neuromuscular activities may be influenced by tDCS.

However, there are still some limitations that should be acknowledged in this study. First, it would be argued that EMG activities of antagonistic muscle in the current study may be influenced by cross-talk contamination, which has been widely concerned in relevant previous researches (Lowery et al., 2003; Farina et al., 2004; Wu et al., 2017). In the current research, an isometric low-force muscle contraction with only a 30% maximal voluntary contraction (MVC) as well as a much smaller size electrode (with a diameter of 6 mm and area of 28 mm^2^) compared to the traditional electrode (with a diameter of 10 mm and area of 79 mm^2^) were adopted, which has been proved to reduce cross-talk effectively (Jaskolska et al., 2006; Farmer et al., 2007; Wang et al., 2020b). Second, a repeated cross-over study design would be better to reduce interference of random factors and increase reliability and generality of the results, which would inevitably increase the cost of the experiment (Machado S. et al., 2019; Machado D. G. D. S. et al., 2019). Third, although participants have stated that they were unable to differentiate tDCS or sham stimulation was applied, potential breaking blinding and/or placebo effects would still play roles and influence the credibility of the results in the current research. However, despite the limitations mentioned above, the current research still gives insights for exploring the influence and mechanism of tDCS on muscular endurance performance. Because the research of exercise training combined with tDCS is still novel, future work should continue to explore the effects of exercise training combined with tDCS and determine the nature of the exercise straining and stimulation intensity dose-response.

## Conclusion

Transcranial direct current stimulation using the Halo Sport device improved muscular endurance performance. The improvement may be partly related to the improvement of neuromuscular efficiency as reflected by decrease of antagonistic muscle coactivation activities, which may be related to cortical originated central processing mechanism of neuromuscular activities.

## Data Availability Statement

The raw data supporting the conclusions of this article will be made available by the authors, without undue reservation.

## Ethics Statement

The studies involving human participants were reviewed and approved by the Ethics Committee of Tongji University. The patients/participants provided their written informed consent to participate in this study.

## Author Contributions

LW, YY, and FZ conceived and designed the study. CW and HY recruited subjects and collected the basic characteristics of subjects. LW, WN, CW, HY, and QS performed the experiments. LW, CW, YY, and FZ made a contribution to data analysis. LW and CW wrote the manuscript. All authors had read and approved the manuscript.

## Conflict of Interest

The authors declare that the research was conducted in the absence of any commercial or financial relationships that could be construed as a potential conflict of interest.

## Publisher’s Note

All claims expressed in this article are solely those of the authors and do not necessarily represent those of their affiliated organizations, or those of the publisher, the editors and the reviewers. Any product that may be evaluated in this article, or claim that may be made by its manufacturer, is not guaranteed or endorsed by the publisher.

## Footnotes

1
http://seniam.org/
